# The importance of HLA DRB1 gene allele to clinical features and disability in patients with multiple sclerosis in Lithuania

**DOI:** 10.1186/1471-2377-13-77

**Published:** 2013-07-09

**Authors:** Renata Balnyte, Daiva Rastenyte, Antanas Vaitkus, Dalia Mickeviciene, Erika Skrodeniene, Astra Vitkauskiene, Ingrida Uloziene

**Affiliations:** 1 Department of Neurology, Academy of Medicine, Lithuanian University of Health Sciences, A. Mickeviciaus street 9, Kaunas LT 44307, Lithuania; 2 Department of Laboratory Medicine, Academy of Medicine, Lithuanian University of Health Sciences, A. Mickeviciaus street 9, Kaunas LT 44307, Lithuania; 3 Department of Otorinolaryngology, Academy of Medicine, Lithuanian University of Health Sciences, A. Mickeviciaus street 9, Kaunas LT 44307, Lithuania

## Abstract

**Background:**

The association of HLA DRB1 alleles with susceptibility to multiple sclerosis (MS) has been consistently reported although its effect on the clinical features and disability is still unclear probably due to diversity in ethnicity and geographic location of the studied populations. The aim of the present study was to investigate the influence of HLA DRB1 alleles on the clinical features and disability of the patients with MS in Lithuania.

**Methods:**

This was a prospective study of 120 patients with MS. HLA DRB1 alleles were genotyped using the polymerase chain reaction.

**Results:**

The first symptoms of MS in patients with HLA DRB1*15 allele manifested at younger age than in those without this allele (28.32 +/− 5.49 yrs vs. 30.94 +/− 8.43 yrs, respectively, p = 0.043). HLA DRB1*08 allele was more prevalent among relapsing-remitting (RR) MS patients than among patients with progressive course of MS (25.0% vs. 8.3%, respectively, chi^2 = 6.000, p = 0.05). MS patients with this allele had lower relapse rate than those without this allele (1.00 +/− 0.97 and 1.44 +/− 0.85, respectively, p = 0.043). Degree of disability during the last visit was lower among the patients with HLA DRB1*08 allele (EDSS score 3.15 +/− 1.95 vs. 4.49 +/− 1.96, p = 0.006), and higher among those with HLA DRB1*15 allele (EDSS score 4.60 +/− 2.10 vs.4.05 +/− 1.94, p = 0.047) compared to patients without these alleles but there were no significant associations between these alleles and the duration of the disease to disability. HLA DRB1*08 allele (OR = 0.18, 95% CI 0,039-0,8, p = 0.029) was demonstradet to be independent factor to take a longer time to reach an EDSS of 6, while HLA DRB1*01 allele (OR = 5.92, 95% CI 1,30-26,8, p = 0.021) was related in a shorter time to reach and EDSS of 6. Patients with HLA DRB1*08 allele had lower IgG index compared to patients without this allele (0.58 +/− 0.17 and 0.73 +/− 0.31, respectively, p = 0.04), and HLA DRB1*15 allele was more often found among MS patients with oligoclonal bands (OCBs) in cerebrospinal fluid than among those without OCBs (OR 2.3, CI 95% 1.017-5.301; p = 0.043).

**Conclusions:**

HLA DRB1*15 allele was related with an earlier manifestation of the first MS symptoms, progressive course of the disease and higher degree of disability. HLA DRB1*08 allele was more prevalent among the RR MS patients and was associated with the lower rate of relapse, degree of disability and IgG index.

## Background

Multiple sclerosis (MS) is a heterogeneous neurodegenerative disease of central the nervous system (CNS), commonly affecting young people and is one of the most common and disabling neurological disorders [1,2]. The importance of human leukocyte antigen (HLA) complex class II genes in the risk to develop MS has widely been studied, but the findings are controversial and might be influenced by the diversity in the ethnicity and geographic location of the studied populations [3,4]. The association between HLA complex genes and the course of MS remains unclear. HLA DRB1*04 was found to be more common among patients with the progressive forms of the disease [5-7]. The studies carried out in the regions with a high prevalence of the HLA DRB1*15 allele showed that this allele was related to the earlier onset of the disease, female sex, and worse health outcomes [5-10]. However, the data are inconsistent: some researchers demonstrated that HLA DRB1*15 was related to a better course and prognosis of MS [9], while others reported that HLA DRB1 alleles had no impact on the severity of the disease, except for a possible relation between HLA DRB1*15 and younger age at the onset of MS [11]. Lithuania belongs to the region with a high prevalence of MS [12]. An association between the HLA DRB1*15 and HLA DRB1*08 alleles and MS was demonstrated among Lithuanian MS patients [13]. Since a geographic region and ethnicity may have a certain impact on the immunogenetic features of the disease, the aim of the present study was to analyze the significance of HLA DRB1 allelic groups to the clinical features and disability of MS patients in Lithuania.

## Methods

### Enrolment of the patients

Altogether, 120 MS patients who were referred to the Department of Neurology, Hospital of Lithuanian University of Health Sciences, in Kaunas between 2009 and 2010, were older than 18 years, and signed written informed consent were enrolled into the study. The diagnosis of MS was established according to the widely accepted and revised McDonald criteria (2005) [14]. The clinical course was the relapsing-remitting (RR) in 60; secondary progressive (SP), in 48; and primary progressive (PP), in 12 patients. All the clinical findings, laboratory data (oligoclonal bands [OGBs] status), magnetic resonance imaging (MRI) findings, and data of visual evoked potentials (VEPs) were reviewed retrospectively from the medical records of the patients. Lumbar puncture and cerebrospinal fluid examination were performed at the time of diagnosis. All imaging studies were conducted with a 1.5-T MR scanner (MAGNETOM Avanto, Siemens, Erlangen, Germany) with a standard head coil. The standard pattern-shift VEPs were recorded for all 120 patients. The registration of VEPs was done by the Evoked Potential Navigating System (Bio-Logic System Corp., USA). The responses were considered abnormal if the P100 latency was longer than 114 ms (i.e., 2 SD above the mean) [15]. Matched CSF and plasma samples were analyzed using isoelectric focusing and IgG specific immunofixation to test for the presence of intrathecal specific OCBs and compared directly with the serum samples [16]. OCBs were defined as positive if more than 2 bands were present in the CSF, but absent in the corresponding blood serum [17]. The IgG index was calculated as the ratio of the product of CSF IgG to serum albumin to the product of the serum IgG and CSF albumin. The IgG index was considered high if it was ≥0.70 [16]. Demographic (age at the onset of the first symptoms, gender) and clinical data (disease course and duration of symptoms, disability status), the findings of all paraclinical tests were recorded for all the patients. Disability was evaluated using the Kurtzke Expanded Disability Status Scale (EDSS).

The study was approved by Kaunas Regional Bioethics Committee. Written informed consent was obtained from each patient before enrolment to the study.

### HLA genotyping

Blood samples were obtained from all patients with MS and stored at −20°C. DNA was extracted from blood leukocytes by the standard phenol-chloroform method [18]. DNA was dissolved in sterile double distilled water. HLA DRB1 alleles for MS patients were genotyped using a polymerase chain reaction (PCR) assay with amplification of the second exon of the genes. An amplified product was manually dot blotted onto nylon membranes. Synthetic sequence-specific oligonucleotide probes were 3’-end-labeled with *α*P32-dCTP and used for hybridization followed by stringency washes and autoradiography. HLA DRB1 alleles were genotyped using the PCR assay with sequence specific primers (HLA DRB1*-PCR) supplied by Protrans and following the manufacturer’s recommendations (PROTRANS Medicinische Diagnostische Produkte GmbH, Germany). Each samples were genotyped by a set of 24 PCRs, which resolved HLA DRB1*01, HLA DRB1*03, HLA DRB1*04, HLA DRB1*07, HLA DRB1*08, HLA DRB1*09, HLA DRB1*11, HLA DRB1*12, HLA DRB1*13, HLA DRB1*14, HLA DRB1*15, and HLA DRB1*16. The amplified products were determined by means of agarose gel electrophoresis. Laboratory analysis was carried out in the Laboratory of Clinical Chemistry and Genetics, Hospital of Lithuanian University of Health Sciences.

### Statistical analysis

Analysis of the collected data was performed using the statistical package SPSS version 13.0. Comparisons of means across groups were carried out using the Student *t* test. Parametric statistical methods were used for the normally distributed quantitative data (estimated with the Kolmogorov-Smirnov and Shapiro-Wilk tests), and means and standard deviations were calculated. The *χ*^2^ test was used to compare the qualitative variables and to estimate possible correlations. Odds ratio (OR) with 95% confidence interval (CI) were calculated to estimate associations. Two factors dispersion analysis was used for the two quantitative variables on a quantitative response. Cox logistic regression method was performed to detect independent factors for reaching an EDSS of 6. The determined level of significance α = 0.05 and *P* values lower than 0.05 (*P* < α) were considered statistically significant.

## Results

### Sample characteristics

The MS group comprised 120 patients; there were 44 men (36.7%) and 76 women (63.3%). The mean age of MS patients was 43.75 ± 10.1 years (41.79 ± 9.97 years for men and 44.8 ± 10.05 years for women). The mean duration of symptoms was 11.93 ± 8.0 years. Of the 120 MS patients, 50.0% had the RR course of the disease; 40.0%, the SP course; and 10.0%, the PR course. The disease most frequently manifested by visual symptoms (61.7%), followed by the brainstem (47.5%) and pyramidal symptoms (45.0%) (See Additional file 1: Table S1 for the original data of descriptive characteristics of MS patients).

### Associations of HLA DRB1 alleles with clinical features and disability of MS patients

The first symptoms of MS in the patients with the RR and PP courses of the disease and who had the HLA DRB1*15 and HLA DRB1*13 alleles manifested at younger age than in those without these alleles (28.32 ± 5.49 and 28.64 ± 6.24 years vs. 30.94 ± 8.43 and 33.94 ± 9.25 years, respectively; *P* < 0.05). No significant associations between age at onset of the first symptoms and other HLA DRB1 alleles were observed (data not shown).

The symptoms of brainstem lesions, as the first symptoms, were more frequently observed among the patients with the HLA DRB1*15 allele than those without it (68.4% vs. 31.6%, respectively; *χ*^2^ = 9.146, *P* = 0.008). On the other hand, brainstem dysfunction was very rare in the patients with the HLA DRB1*08 allele compared with those without this allele (8.8% vs. 91.2%, respectively; *χ*^2^ = 4.872, *P* = 0.027). No significant associations were found between other HLA DRB1 alleles and the first symptoms.

The HLA DRB1*08 allele was more frequently documented among the RR MS patients than among the patients with the progressive forms of MS (*P* = 0.014), while the HLA DRB1*15 allele was more prevalent among the patients with the progressive forms of MS (*P* < 0.001) (See Additional file 2: Table S2 for the original data of prevalence of HLA DRB1 alleles in patients with multiple sclerosis according to the course of the disease).

The MS patients with the HLA DRB1*08 allele had a lower relapse rate than those without this allele (1.00 ± 0.97 and 1.44 ± 0.85, respectively, *P* = 0.043). No significant associations were found between the other alleles and the relapse rate (data not shown).

The lowest EDSS score during the last visit was among the patients with the HLA DRB1*08 allele compared with the patients without this allele (3.15 ± 1.95 vs. 4.49 ± 1.96, *P* = 0.006) and the highest one among those with HLA DRB1*15 allele (4.60 ± 2.10 vs. 4.05 ± 1.94, *P* = 0.047), but there were no significant associations between these alleles and the duration of the disease to disability. Other alleles showed no significant relationship with the degree of disability during the last visit (data not show).

In the multivariate analysis, the HLA DRB1*08 allele (OR = 0.18, 95% CI 0.039-0.8, *P* = 0.029) was demonstradet to be independent factor to take a longer time to reach an EDSS of 6, while HLA DRB1*01 allele (OR = 5.92, 95% CI 1.30-26.8, *P* = 0.021) was related in a shorter time to reach and EDSS of 6.

Only HLA DRB1*15 allele was found to be associated with MRI changes in the brainstem (OR = 3.08, 95% CI 2.23-4.25; *P* = 0.001) and abnormal VEPs (OR = 1.50, 95% CI 1.18-2.02, *P* = 0.022).

The patients with the HLA DRB1*08 allele had a lower IgG index compared with the patients without this allele (0.58 ± 0.17 and 0.73 ± 0.31, respectively; *P* = 0.04). The patients with the HLA DRB1*15 allele had a higher IgG index than those without this allele; however, the difference was not statistically significant (0.72 ± 0.30 vs. 0.67 ± 0.29, respectively; *P* = 0.3). The HLA DRB1*15 allele was more common among the MS patients with OGBs in the cerebrospinal fluid than those without OGBs in the cerebrospinal fluid (80.6% vs. 64.2%; OR = 2.3, 95% CI 1.017-5.301; *P* = 0.043) (See Additional file 3: Figure S1 for the original data of association of HLA DRB1*15 allele with oligoclonal bands).

No associations were found between the other HLA DRB1 alleles and OCBs as well as IgG index in MS patients.

## Discussion

A strong genetic association between the HLA-DRB1 genotype and MS susceptibility has consistently been demonstrated across populations [2,5,8,9,19], but data on the association between this genotype and the severity and outcome of the disease are controversial [6,8,11,19-21]. We have previously shown that the HLA-DRB1*15 and *08 alleles are strongly associated with disease risk in the Lithuanian population (13). Lithuania is in the North-eastern European region, and its population has a pure gene pool [22]; therefore, the results of the analysis of immunological and genetic factors and their influence on the MS course may be valuable to scientists and physicians not only in Lithuania, but in other countries as well. The results of our study indicate that the patients with progressive MS had the HLA DRB1*15 allele more frequently, and this allele was related to younger age of the first symptoms and greater disability. Meanwhile, the HLA DRB1*08 allele was more common in the patients with relapsing-remitting MS, and these patients had a lower degree of disability. The HLA DRB1*15 allele was more common among the MS patients who had OCBs in the cerebrospinal fluid. A lower IgG index was documented in the patients with the HLA DRB1*08 allele than those without this allele.

Controversial and discrepant views exist regarding the associations between HLA DRB1 alleles, especially HLA DRB1*15, and the onset, progression, or severity of the disease. Several authors have suggested that the HLA DRB1*15 allele might be associated with younger age at the onset of MS and worse prognosis of the disease [6,8,11,20,23]. In the present study, not only HLA DRB1*15, but also HLA DRB1*13, was found to be associated with younger age at the onset of MS. Contrary to our data, one Portuguese study demonstrated HLA DRB1*15 to be related to a better prognosis of MS [19], while others researchers failed to prove it as a prognostic factor [5,9,24]. The HLA DRB1*04 and HLA DRB1*01 alleles have also been reported to be associated with a worse prognosis of MS [5-7,21]. In our study, the HLA DRB1*01 allele was related in a shorter time to reach and EDSS of 6, this likely to be related to a worse prognosis. Moreover, the relationship of the HLA DRB1*15 allele with MRI changes in the brainstem supports the opinion that this allele might be associated with a worse prognosis of the disease. Our findings corroborate the results of one study, where MS lesions, cerebral atrophy, and cognitive impairment were more common among patients carrying this allele [10]. However, other study showed that HLA DRB alleles, especially HLA DRB1*15, did not have any impact on the development of cerebral atrophy and cognitive dysfunction [11]. The detection of the HLA DRB1*04 and *09 alleles might be related to a greater number of MRI changes and more rapid progression of disability [25], while in our study, these alleles, especially HLA DRB1*09, appeared to be observed quite rare and had no influence on the clinical signs of MS [13]. The HLA-DRB1*0801 allele was associated with older age at onset [23,26], but in the present study, this allele was related to a relatively better course of the disease and less severe disability.

In our study, the HLA DRB1*15 allele was found in 80.6% of MS patients having OCBs in their cerebrospinal fluid. These findings are in line with the results of other studies from Australia [26], Turkey [27], and Spain [28] demonstrating that a higher frequency of the HLA DRB1*15 allele could be associated with OCBs. Australian researchers in their cohort trial demonstrated that the presence of OCBs might also be related to the HLA DRB1*03 and *04 alleles [29]. We failed to confirm such a relation in the present study. An association between OCBs and the HLA DRB1*15 allele suggests that immunological changes affecting the prognosis of the disease might be regulated by genetic factors, i.e., supports an idea of genetic origin of MS. Our findings also suggest that the presence of OCB is associated with the HLA DRB1 genotype and that different genotypes are linked to the different rates of intrathecal immunoglobulin synthesis, in turn reflecting the levels of B cell activity in the CNS [30].

Our study has several limitations that have to be mentioned. First, although it was a prospective study, some clinical data were gathered retrospectively from the medical records. Second, the data presented here were collected from a relatively small sample of MS patients and this could lead to partly inconclusive results. These results have to be confirmed in a larger cohort of Lithuanian MS patients.

## Conclusions

A certain association between HLA DRB1 alleles and the course of disease has been established among Lithuanian patients. The HLA DRB1*15 allele was found to be related to younger age of the first symptoms, progressive course of the disease, and a higher degree of disability, while the HLA DRB1*08 allele was more common in the patients with relapsing-remitting MS and a lower degree of disability. Our results suggest that HLA DRB1 alleles may have an impact on the clinical manifestation of MS, natural course of the disease and thus provide with some additional information on prognosis and future perspectives to a patient; however, further and large-scale studies are needed.

## Competing interests

The authors declare that they have no competing interests.

## Authors’ contributions

RB performed clinical investigations of MS patients, acquired the data, contributed to analysis and interpretation of the data, and drafted the initial version of the manuscript. AV and ES performed and evaluated genetic and immunological studies. DM, IU, DR, and AV were involved in revising the manuscript and gave the final approval to the version to be published. All the authors read and approved the final manuscript.

## Pre-publication history

The pre-publication history for this paper can be accessed here:

http://www.biomedcentral.com/1471-2377/13/77/prepub

## Supplementary Material

Additional file 1: Table S1 The main demographical and clinical data of the multiple sclerosis patients. Click here for file

Additional file 2: Table S2 Prevalence of HLA DRB1 alleles in patients with multiple sclerosis according to the course of the disease. Click here for file

Additional file 3: Figure S1 The original data of association of HLA DRB1*15 allele with oligoclonal bands. Click here for file
