# Supplementary material for: The importance of HLA DRB1 gene allele to clinical features and disability in patients with multiple sclerosis in Lithuania
Source: BMC Neurol. 2013 Jul 9;13:77. doi: 10.1186/1471-2377-13-77 (PMC3716946; doi:10.1186/1471-2377-13-77)
Supplement: Additional file 1: Table S1 — The main demographical and clinical data of the multiple sclerosis patients. [file 1471-2377-13-77-S1.docx]

Additional file 1 – The main demographical and clinical data of the multiple sclerosis patients (Table 1. docx)

**Table 1.** The main demographical and clinical data of the multiple sclerosis patients

| **Characteristic** | **MS patients**  **N = 120** |
| --- | --- |
| Gender ratio (M:F) | 1:1.72 (44:76) |
| Age at onset, years, mean ± SD (range) | 30.86± 7.92 (16- 55) |
| Disease course**:** | |
| Relapsing-remitting | 60 (50.0%) |
| Secondary-progressive | 48 (40.0%) |
| Primary-progressive | 12 (10.0%) |
| Duration of the symptoms, years, mean ± SD | 11.93 ± 8.0 |
| Duration of the disease (time of diagnosis), years, mean± SD | 6.84 ± 3.54 |
| Relapse rate per year, mean ± SD | 1.36 ± 0.88 |
| First symptoms**:** | |
| Pyramidal | 54 (45.0%) |
| Sensory | 42 (35.0%) |
| Visual | 74 (61.7%) |
| Blower/bladder | 20 (16.7%) |
| Cerebellar | 43 (35.8%) |
| Brainstem | 57 (47.5%) |
| Mental | 40 (33.3%) |
| EDSS^A^ score, mean ± SD | 4.26 ± 2.01 |
| EDSS^B^ score, mean ± SD | 3.8 ± 1.0 |
| MRI lesions**:** |  |
| Periventricular | 116 (96.7%) |
| Corpus callosum | 104 (86.7%) |
| Brainstem | 77 (64.2%) |
| Cerebellum | 59 (49.2%) |
| Juxtacortical | 63 (52.5%) |
| Spinal cord | 25 (20.8%) |
| VEP abnormalities OS/OD (n, %) | 85 (70.8%)/ 83 (69.2%) |
| IgG index  OCBs (n, %) | 0.70 ± 0.29  88 (73.3%) |

Abbreviations: *SD - standard deviation, EDSS - Expanded Disability Status Scale , EDSS^A^ - EDSS score during last visit, EDSS^B^ - EDSS score at a time of diagnosis, MRI – Magnetic Resonance Imaging, VEP - Visual Evoked Potential, OS- oculus dextri, OS- oculus sinistri, IgG index- Immunoglobulin G index, OCBs- oligoclonal bands.*
