# Supplementary material for: The importance of HLA DRB1 gene allele to clinical features and disability in patients with multiple sclerosis in Lithuania
Source: BMC Neurol. 2013 Jul 9;13:77. doi: 10.1186/1471-2377-13-77 (PMC3716946; doi:10.1186/1471-2377-13-77)
Supplement: Additional file 2: Table S2 — Prevalence of HLA DRB1 alleles in patients with multiple sclerosis according to the course of the disease. [file 1471-2377-13-77-S2.docx]

Additional file 2 – Prevalence of HLA DRB1 alleles in patients with multiple sclerosis according to the course of the disease (Table 2. docx).

*Table 2. Prevalence of HLA DRB1 alleles in patients with multiple sclerosis according to the course of the disease*

| HLA DRB1 alleles | MS course | | | *χ²* | p  value |
| --- | --- | --- | --- | --- | --- |
|  | RR MS  N = 60 | SP MS  N= 48 | PP MS  N= 12 |  |  |
| *01 n (%) | 2 (3.3) | 6 (12.5) | 1 (8.3) | 3.243 | 0.2 |
| * 03 n (%) | 4 (6.7) | 4 (8.3) | 2 (16.6) | 1.309 | 0.5 |
| * 04 n (%) | 7 (11.7) | 9 (18.8) | 2 (16.6) | 1.078 | 0.58 |
| * 07 n (%) | 15 (25.0) | 12 (25.0) | 3 (25.0) | 0.001 | 0.9 |
| * 08 n (%) | 15 (25.0) | 4 (8.3) | 1 (8.3) | 6.000 | 0.05 |
| * 11 n (%) | 16 (26.7) | 10 (20.8) | 1 (8.3) | 2.055 | 0.35 |
| * 12 n (%) | 5 (8.3) | 3 (6.2) | 1 (8.3) | 0.180 | 0.9 |
| * 13 n (%) | 9 (15.0) | 8 (16.7) | 2 (16.6) | 0.063 | 0.9 |
| * 14 n (%) | 2 (3.3) | 0 (0.0) | 1 (8.3) | 3.077 | 0.2 |
| * 15 n (%) | 26 (43.3) | 32 (66.7) | 10 (83.3) | 5.347 | 0.076 |
| * 16 n (%) | 3 (5.0) | 1 (2.1) | 0 (0.0) | 1.164 | 0.5 |

*Abbreviations:* *RR – relapsing-remitting, SP – secondary progressive, PP – primary progressive, χ²* - *chi-square test.*
