# Supplementary material for: The importance of HLA DRB1 gene allele to clinical features and disability in patients with multiple sclerosis in Lithuania
Source: BMC Neurol. 2013 Jul 9;13:77. doi: 10.1186/1471-2377-13-77 (PMC3716946; doi:10.1186/1471-2377-13-77)
Supplement: Additional file 3: Figure S1 — The original data of association of HLA DRB1*15 allele with oligoclonal bands. [file 1471-2377-13-77-S3.docx]

Additional file 3 - The original data of association of HLA DRB1*15 allele
with oligoclonal bands (Fig 1. ppt).

***Fig 1.*** *Association of HLA DRB1*15 allele
with oligoclonal bands (P = 0.043)*
